# Effect of Oxygen Plasma on Sprout and Root Growth, Surface Morphology and Yield of Garlic

**DOI:** 10.3390/plants8110462

**Published:** 2019-10-30

**Authors:** Matej Holc, Gregor Primc, Jernej Iskra, Primož Titan, Janez Kovač, Miran Mozetič, Ita Junkar

**Affiliations:** 1 Jozef Stefan Institute, Jamova 39, 1000 Ljubljana, Slovenia; 2 Jozef Stefan International Postgraduate School, Jamova 39, 1000 Ljubljana, Slovenia; 3 RGA d.o.o., Brodarska 27, Krog pri Murski Soboti, 9000 Murska Sobota, Slovenia; 4 Faculty of Chemistry and Chemical Technology, Večna pot 113, 1000 Ljubljana, Slovenia

**Keywords:** garlic, oxygen plasma, growth, cuticle, water uptake, nanostructure, yield

## Abstract

Depending on the climate, garlic can be planted either in the fall or spring for a harvest in the summer, but spring planting might require the strengthening of the plant by external techniques. We have used low pressure, inductively coupled, radio frequency oxygen plasma for the treatment of peeled garlic cloves of a spring-planted Slovenian autochthonous cultivar. The aim of the study was to assess the effects of plasma treatment on garlic clove shoot and root growth and, ultimately, the yield. The roles of surface chemistry, surface morphology, and water uptake in these effects were also evaluated. The plasma treatment of cloves induced increases in water uptake. The increases were explained by changes in surface morphology that were determined by using scanning electron microscopy (SEM) and atomic force microscopy (AFM). Nanostructured epicuticular wax structures appeared at the cuticle surface. The optimal treatment parameters accelerated root growth, but not shoot growth, in a laboratory setting. After growth in the field, the trends indicated that plant height and dried bulb mass increase, but the improvements were not statistically significant.

## 1. Introduction

The presence and availability of local, autochthonous garlic (*Allium sativum*) cultivars is commonly threatened by imported varieties. In Slovenia, this holds true for the autochthonous variety *Ptujski spomladanski* (Ptuj spring garlic), which might be either fall- or spring-planted. If planted in the spring, garlic lacks the advantage of starting its development in the fall and, thus, requires early planting to properly develop its root system in time. Further, it might lack the low temperature exposure that is required to break dormancy and initiate clove formation and bulbing [1]. Planting at the optimal time is often hindered by external conditions, such as wet soil or freezing of the soil due to low temperatures, as weather conditions are becoming increasingly unpredictable. Therefore, it is desired to strengthen the garlic plant and improve its germination, in particular to stimulate and accelerate the growth of roots, so that a proper root system might be established and the bulbs may grow efficiently, which leads to high yields.

In an attempt to avoid the excess use of fertilizers or chemical growth stimulants to improve plant performance in the field, plasma agriculture has emerged as an environmentally acceptable alternative. The use of non-thermal plasma in agriculture is ecologically benign, does not introduce toxic compounds into the environment, and does not damage the sensitive planting material. So far, the gas plasma treatment of various agricultural seeds has been shown to affect many stages of plant germination and growth [2], including shoot and root growth of seedlings. At atmospheric pressure, dielectric barrier discharges (DBD) in air have increased the shoot and root length of wheat [3,4], pea [5], and root length of maize [6], while using argon increased shoot and root length of pepper [7], and nitrogen and helium improved the same parameters for wheat [8]. Regarding plants of the genus *Allium*, Pawłat et al. have treated onion seeds with a radio frequency (RF) plasma jet, which results in increased seedling length [9]. While many studies using low-pressure plasma have focused on germination rate of seeds, some have also reported improvements in other parameters. Seed treatment in a RF capacitively coupled plasma (CCP) using helium increased shoot and root length of soybean [10] and rapeseed [11], as well as root length of tomato [12], while the use of nitrogen increased root length and weight of artichoke [13].

Further, the planting of plasma treated seeds can result in improved yield of important agricultural crops. An increase in wheat yield was achieved by the use of low-pressure helium plasma [14], low pressure air plasma [15], and medium pressure air and air/oxygen plasma [16], whereas tomato yield could be improved by atmospheric pressure air plasma, either an arc discharge [17] or a dielectric barrier discharge (DBD) [18]. A low pressure, helium plasma improved peanut yield [19], while the seed yield of *Arabidopsis thailana* was improved by an atmospheric pressure air DBD [20]. The effect of plasma on both germination and yield varies with plasma parameters, as well as plant species, and it should be optimized for each specific case. To the best of our knowledge, this is the first study regarding the yield of garlic after the use of plasma treatment.

Most agriculturally produced garlic is sterile and grown from cloves, a vegetative kind of plant propagation material [1]. In parallel with findings regarding the germination of seeds, we have treated garlic cloves with weakly ionized low-pressure oxygen plasma. We have worked with the Slovenian autochthonous garlic cultivar *Ptujski spomladanski*, which can be spring-planted. The aim of the present study was to record the effects on garlic shoot and root growth and, ultimately, the yield, after exposure of cloves to low-pressure oxygen plasma. It was of particular interest to link any improvements in root growth to eventual yield improvement, as well as relate the growth of garlic to plasma-induced changes in morphology and physico-chemical properties of the garlic clove surface.

## 2. Materials and Methods

### 2.1. Garlic

The garlic seed bulbs of a Slovenian autochthonous cultivar, *Ptujski spomladanski*, were purchased from Semenarna Ljubljana, Slovenia, and stored at suitable conditions. Immediately before plasma treatment, the bulbs were separated into cloves, which were peeled.

### 2.2. Plasma Treatment

**Figure 1:**
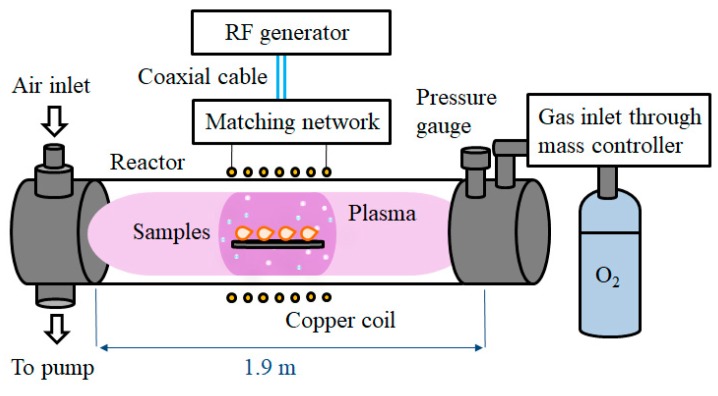
Schematic representation of the plasma system used in the treatment of garlic cloves.

We have chosen an in-house designed, large-scale plasma system for the treatment of garlic cloves while using a low pressure, inductively coupled, RF oxygen plasma. The reactor of the plasma system is a borosilicate discharge tube that is approximately 2000 mm in length and 200 mm in diameter. To sustain the desired low pressure, the reactor is continuously pumped while using a two stage oil rotary vacuum pump of the nominal pumping speed of 80 m^3^/h that was connected to one end of the reactor. A simple air inlet valve for venting the whole vacuum system is mounted on the same side. Commercially available oxygen of 99.99% purity is introduced at the opposite end of the tube by a flow controller AERA PC7700 (Advanced Energy, Fort Collins, CO, USA) that was calibrated for oxygen at 20 °C. On the same side, a Baratron absolute pressure gauge (MKS Instruments, Andover, MA, USA) is mounted to monitor the pressure inside the reactor. A double copper coil, one four-, and one five-turn, is wrapped around the tube, and an RF power generator (Induktio d.o.o., Litostrojska 44d, Ljubljana, Slovenia) operating at 27.12 MHz is coupled to the coil via a matching network. Figure 1 illustrates a schematic representation of the system.

During plasma treatment, the coil was positioned in the center of the tube. The plasma intensity is highest inside the coil, but capacitive coupling is present throughout the tube. The cloves were placed on a glass plate and positioned inside the coil. One to five cloves were simultaneously treated. The plasma was ignited at a pressure of 15–60 Pa at a forward power of approximately 400 W. Treatment time, as selected during preliminary experiments, was 60 s. During this time, the temperature of the cloves did not perceivably increase.

### 2.3. X-ray Photoelectron Spectroscopy (XPS)

An XPS instrument (Physical Electronics, Ismaning, Germany) was used to obtain the elemental composition of the clove surface. Pieces that were approximately 4 mm × 4 mm in size and 1 mm in thickness were excised from treated and untreated cloves using a scalpel, and then freeze dried. The samples were mounted onto an XPS sample holder, and their surfaces were excited by X-ray radiation from a monochromatic Al source at photon energy of 1486.6 eV. Each sample was measured three times and the mean chemical composition was calculated. The high-energy resolution spectra were acquired with an energy analyzer operating at resolution of about 0.5 eV and a pass energy of 29 eV. The spectra were analyzed using the MultiPak V8.0 software (Ulvac-phi, Inc., Chigasaki, Japan). They were calibrated while using a C 1s peak at 284.8 eV.

### 2.4. Atomic Force Microscopy (AFM)

An AFM (Solver PRO, NT-MDT, Moscow, Russia) was used to characterize the topology of the samples. Pieces approximately 4 mm × 4 mm in size and 1 mm in thickness were excised from the surface of treated and untreated cloves while using a scalpel and mounted onto aluminum holders using carbon tape. All of the measurements were performed in tapping mode using ATEC-NC-20 tips (Nano and More GmbH, Wetzlar, Germany) with a resonance frequency of 210–490 kHz and the force constant of 12–110 N/m.

### 2.5. Scanning Electron Microscopy (SEM)

For SEM analysis, pieces of approximately 4 mm × 4 mm in size and 1 mm in thickness were excised from the surface of treated and untreated cloves while using a scalpel and then freeze dried. The samples were attached onto aluminum stubs using conductive carbon tape and coated with a thin layer of platinum using a Gatan 682 Precision Etching and Coating System (Gatan Inc., Pleasanton, California). The SEM images were obtained using a JSM-7600F Schottky Field Emission SEM (Jeol Ltd., Tokyo, Japan).

### 2.6. Water Uptake (WU)

To determine WU, for each condition, five separately treated cloves were submerged into a beaker with 100 mL DI water immediately after treatment. After 30 min., the cloves were removed from the beakers, excess water was wiped off, and the cloves were weighed. WU was calculated according to the equation

(1) $$m=\frac{\left(m_{30}-m_{0}\right)}{m_{0}}\times 100\%,$$

where m is the increase in mass, expressed as percentage of m_0_, while m_0_ and m_30_ represent the clove mass immediately before and after 30 minutes of soaking, respectively. The 30 min. soaking time was selected in preliminary experiments as a balance between WU increase and duration of the experiment.

### 2.7. Shoot and Root Growth

To sprout cloves in a laboratory setting, each clove was placed on filter paper inside a petri dish and moistened with 1 mL of DI water. The cloves were incubated at room temperature in the dark; 0.5 mL of DI water was added as needed. 20 cloves (four replications of five cloves each) were used for each treatment condition.

Shoot length was recorded up to 30 days after incubation; the shoots were measured from the lowest point of emergence from the garlic storage leaf up to the tip. The length of the longest root and total number of roots were recorded up to 15 days after incubation, after which, the roots began to decay in the laboratory setting. In case of decayed cloves or roots, the last recorded observation was used in place of missing data for calculation of means.

Two control samples were included in addition to the untreated control: a vacuum control, where the cloves were exposed to low pressure and gas flow, and an electromagnetic (EM) field control, where they were exposed to the induced EM field of the plasma outside of the plasma reactor. Both of the controls showed no significant differences in results as compared to the untreated control.

### 2.8. Plant Height and Yield

The cloves were planted in a field close to Ptuj, Slovenia, one day after plasma treatment. 15 cloves per treatment condition (three replications of five cloves each) were used to grow garlic from treated and untreated cloves. They were planted in the spring (March 2018) and harvested after approximately four months (July 2018), when the plant height was recorded from the bottom of the bulb to the top of the longest leaf. The garlic was dried for a month, after which, it was cleaned, leaves and roots were removed, and mass of each bulb was recorded. Mean height and dry bulb mass were calculated.

### 2.9. Statistical Analysis

Data from the WU, shoot and root growth, plant height, and yield measurements were statistically analyzed while using the JASP 0.9.2. open-source software (University of Amsterdam, Amsterdam, The Netherlands). Group means were calculated and compared using ANOVA, followed by the post hoc Tukey’s range test. Means were considered significantly different at (*p*- < −0.05).

## 3. Results

### 3.1. Surface Chemistry

**Figure 2:**
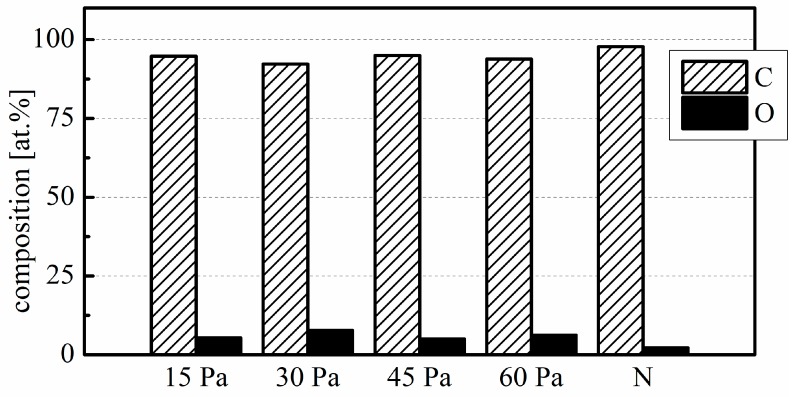
Elemental composition of the garlic clove surface. 15 Pa, 30 Pa, 45 Pa, 60 Pa: plasma treatments at the indicated working pressure; N: untreated control.

As seen in the XPS results in Figure 2, the untreated clove surface consists of about 98 at.% carbon (C) and about 2 at.% oxygen (O). After plasma treatment, the O content increases to a total of about 5–8 at.%, and C content decreases accordingly.

Table 1 shows the results of high-energy resolution C 1s spectra peak fitting. The low initial relative concentrations of C–O and O–C=O bonds are increased with plasma treatment, but there is a lack of clear correlation between relative concentration and working pressure.

### 3.2. Surface Morphology

**Figure 3:**
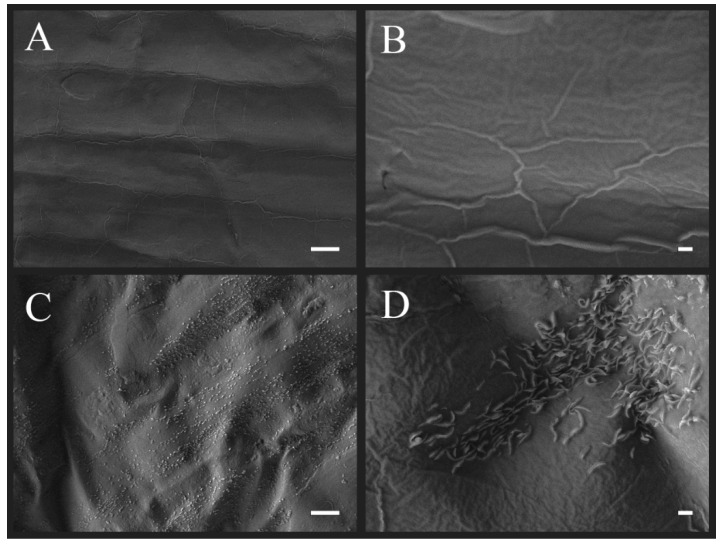
Scanning Electron Microscopy (SEM) images of garlic clove surface. (**A**,**B**): untreated control; (**C**,**D**): plasma treatment at 30 Pa. (**A**,**C**): 1000× magnification, scale bar = 10 µm; and, (**B**,**D**): 5000× magnification, scale bar = 1 µm.

**Figure 4:**
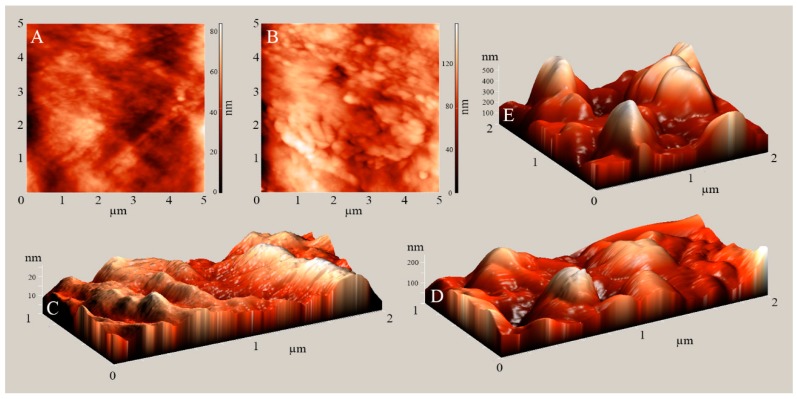
Atomic Force Microscopy (AFM) images of garlic clove surface, showing an area of 5 × 5 µm (**A**,**B**), 2 × 1 µm (**C**,**D**), or 2 × 2 µm (**E**). (**A**,**C**): untreated control; (**B**,**D**): plasma treatment at 30 Pa; (**E**): wax platelets visible on the sample treated at 30 Pa.

SEM images (Figure 3) and AFM scans (Figure 4) reveal a surface of untreated cloves that is wrinkled, but reasonably smooth. However, large patches of plasma treated surface are more intensely wrinkled than the untreated one, which indicates a degree of etching or reshaping. In addition, wax structures appear at the clove surface. The size of the structures is approximately up to 1 µm in length and up to 100 nm in width. We estimate that their height is up to a few hundred nm. In a SEM overview of treated clove surface, we have seen that the structures appear in discreet areas, which are reminiscent of islands or stripes. While it is difficult to estimate the total covered area, it appears to increase with decreasing pressure, which suggests that the structures are more abundant after treatment with a higher reactive species dose. The density of the structures appears uniform in areas where they are present. 

### 3.3. Water Uptake

**Figure 5:**
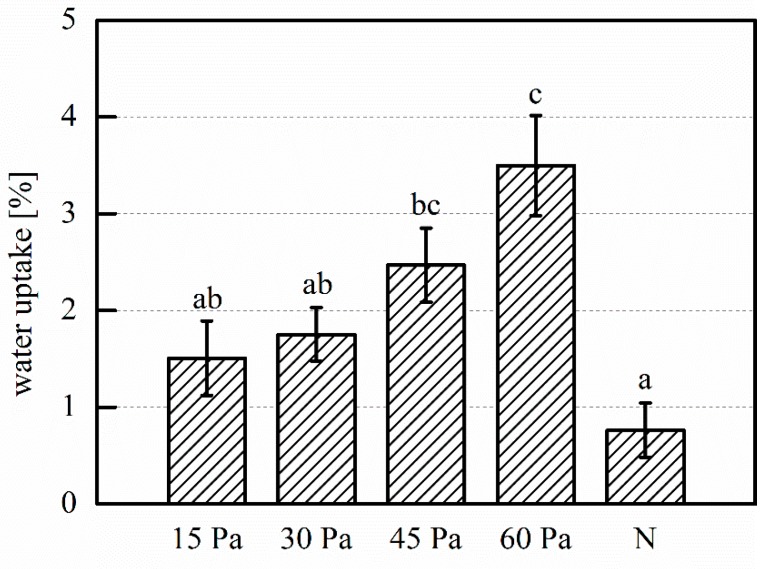
Mean mass increase of the clove after 30 minutes of soaking. 15 Pa, 30 Pa, 45 Pa, 60 Pa: plasma treatments at the indicated working pressure; N: untreated control. Number of replications: n = 5. Error bars indicate standard error. Different letter labels indicate statistically significant differences (*p* < 0.05).

After soaking in deionized (DI) water, an increase of WU is seen with the increase of working pressure, as shown in Figure 5. The WU increases for treatments at 45 and 60 Pa are significant when compared to the untreated control. WU for the 60 Pa treatment is further significantly larger than the WU for the 15 and 30 Pa treatments.

### 3.4. Shoot and Root Growth

**Figure 6:**
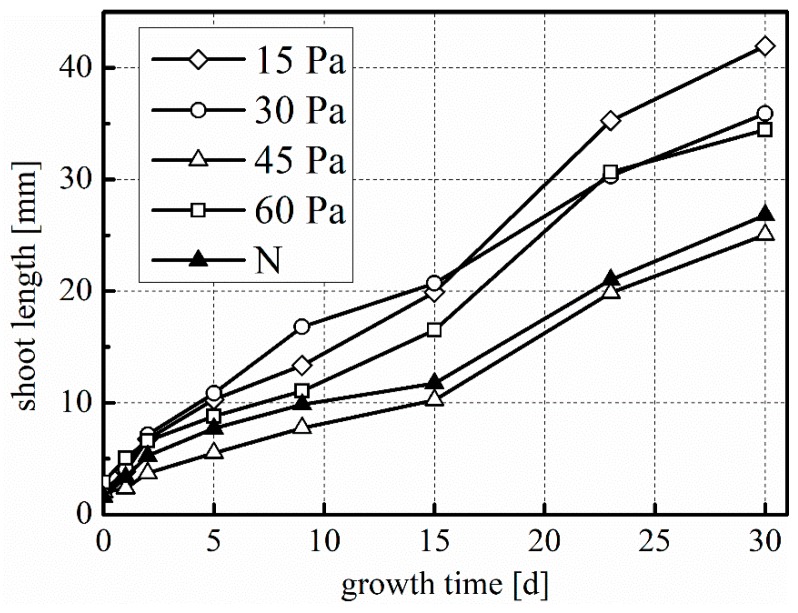
Mean shoot growth during laboratory germination. 15 Pa, 30 Pa, 45 Pa, 60 Pa: plasma treatments at the indicated working pressure; N: untreated control. Number of replications: n = 4 (of five samples each, total 20 samples).

**Figure 7:**
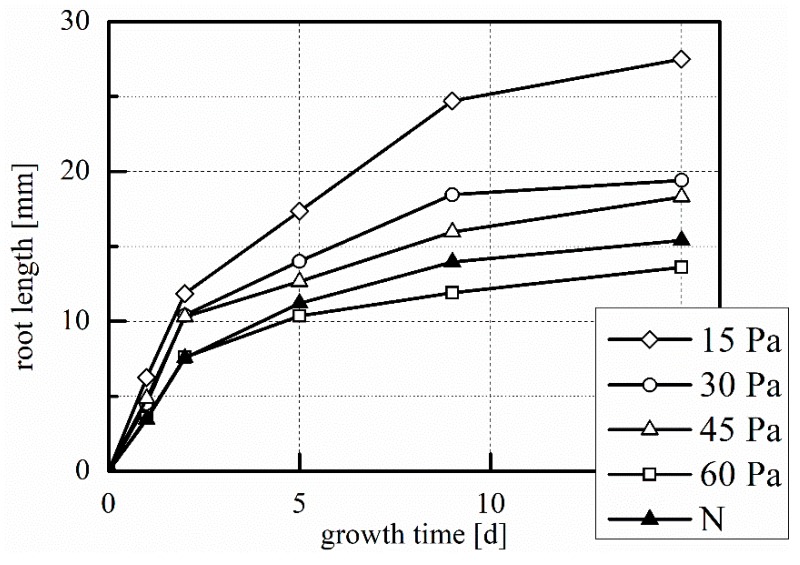
Mean root growth during laboratory germination. 15 Pa, 30 Pa, 45 Pa, 60 Pa: plasma treatments at the indicated working pressure; N: untreated control. Number of replications: n = 4 (of 5 samples each, total 20 samples).

**Figure 8:**
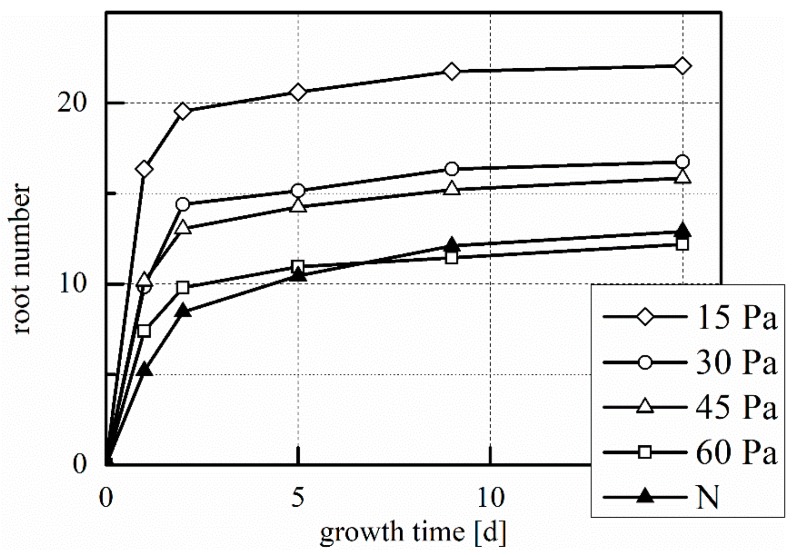
Mean root number during laboratory germination. 15 Pa, 30 Pa, 45 Pa, 60 Pa: plasma treatments at the indicated working pressure; N: untreated control. Number of replications: n = 4 (of five samples each, total 20 samples).

As seen in Figure 6, plasma treatment has somewhat accelerated shoot growth, which is comparable for all treatments except 45 Pa. The improvements are not statistically significant. With a decrease in working pressure, we also see a trend of increase in both root length (Figure 7) and root number (Figure 8). For the treatment at 15 Pa, the results are significantly greater than the untreated control and the 60 Pa treatment, which is true for root length at t = 5, 9, and 15 days, and for root number at all data points, except t = 0 days.

### 3.5. Plant Height and Yield

**Figure 9:**
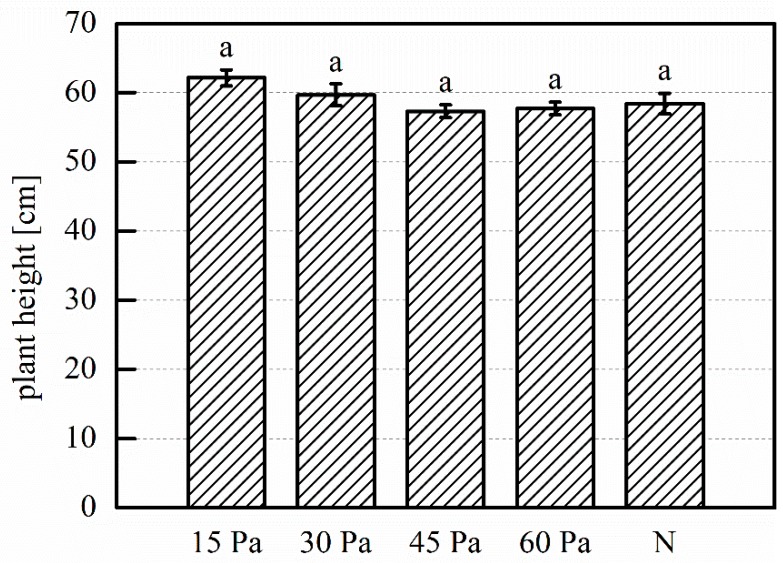
Mean plant height at harvest of garlic plants grown from treated and untreated cloves. 15 Pa, 30 Pa, 45 Pa, 60 Pa: plasma treatments at the indicated working pressure; N: untreated control. Number of replications: n = 3 (of five samples each, total 15 samples). Error bars indicate standard error. Different letter labels indicate statistically significant differences (*p* < 0.05).

**Figure 10:**
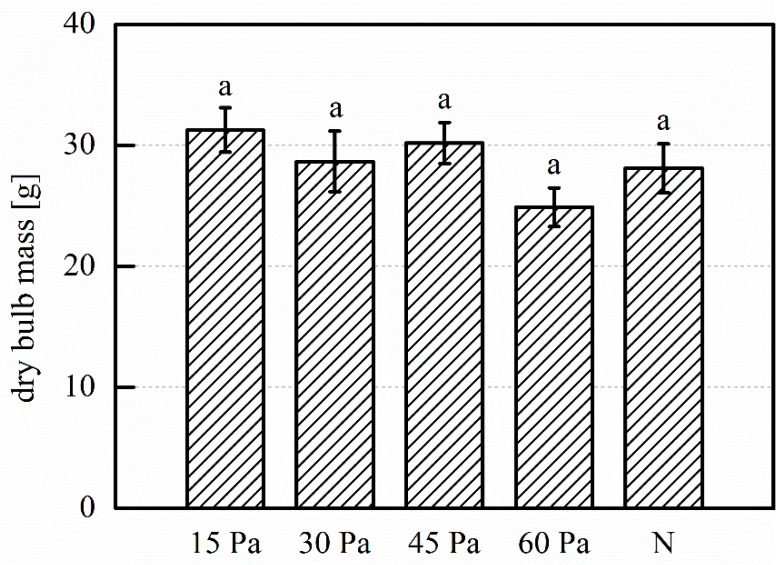
Mean mass of dried garlic bulbs of garlic plants grown from treated and untreated cloves. 15 Pa, 30 Pa, 45 Pa, 60 Pa: plasma treatments at the indicated working pressure; N: untreated control. Number of replications: n = 3 (of five samples each, total 15 samples). Error bars indicate standard error. Different letter labels indicate statistically significant differences (*p* < 0.05).

Regarding plant height at harvest, in Figure 9 we see a modest increase for the 15 Pa treatment. The remaining treatments compare to the control, and a slight trend of height decrease with increasing pressure is present. The increases are not statistically significant. In Figure 10, an 11% increase of dried bulb mass is seen for the 15 Pa treatment. Very limited increases are also seen for the 30 Pa and 45 Pa treatments, while the dried mass for the 60 Pa treatment is decreased. The differences are not statistically significant.

## 4. Discussion

The bulk of the peeled garlic clove is technically a storage leaf and, like many plant surfaces, it is covered by a cuticle [21]—a protective layer of hydrocarbon polymers impregnated and covered with wax. Its outer layer, epicuticular wax, typically comprises an array of saturated long-chain hydrocarbons, with or without a terminal O-containing functional group [22]. This is reflected in the XPS analysis result of the untreated clove surface, which contains a few at.% of O in addition to C (Figure 2). During plasma treatment, reactive oxygen species (ROS) chemically react with the clove surface, which results in oxidation, and thereby a modestly increased O concentration. In high-energy-resolution spectra of C 1s (Table 1), the main peak, at a binding energy of ~284.8 eV, corresponds to C–C/C–H bonds, which represent the majority of C bonds in the predominantly hydrocarbon composition of epicuticular wax. As expected, we recorded increases in the relative concentrations of C–O and O–C=O bonds after plasma treatment, as denoted by increases in the area of sub-peaks at ~286.5 and ~289.0 eV, respectively. The lack of correlation between the relative concentration increases and working pressure is due to the limited nature of the changes and non-uniformity of clove surface chemistry. Nonetheless, the C–O and O–C=O sub-peak area increases indicate oxidation of the wax hydrocarbons, as well as their oxygen-containing functional groups, expected to progress from hydroxyl to carbonyl, and finally to carboxyl groups [23]. In our previous work on unpeeled garlic cloves, which have a similar surface elemental composition when untreated, exposure to low-pressure oxygen plasma afterglow in a small-scale reactor has produced a significantly higher increase in surface O content, up to about 20 at.% after as little as 15 s of treatment [24]. This is the consequence of the higher reactive species density in that experiment due to the higher power density in the smaller reactor volume. In the present case, the analysis also revealed no presence of mineral elements (silicon, calcium, potassium), which indicates that no substantial etching of the surface took place during plasma treatment.

Even though numerous plant species also exhibit cuticles as the outer layers of their seed coats [25], direct comparison to garlic cloves is difficult due to differences in seed coat structure and composition. While using XPS, untreated surface of quinoa seeds was found to contain about 89 at.% C and 10 at.% O, while, after air plasma treatment, either atmospheric pressure DBD or low pressure RF, the O content increased to about 25–30 at.% [26]. In another study, atmospheric air surface DBD increased the surface O content from about 20 at.% to about 40 at.% for cucumber and pepper seeds [27]. Further, alteration of surface chemistry after plasma treatment depends on the process gas used and, to the best of our knowledge, no study so far has analyzed the seed surface chemistry after treatment with pure oxygen plasma.

The observed morphological changes are quite prominent, despite the limited change in surface chemistry seen in XPS after plasma treatment. As seen in Figure 3, the untreated cuticle appears as a wavy, but flat film. After plasma treatment, protruding wax structures appear at the surface. On many plant cuticle surfaces, epicuticular waxes readily self-assemble into crystalline structures. Their morphology varies widely; it is associated with plant species and determined by the chemical constituents of the wax. If such wax is deposited on an artificial surface after extraction or evaporation, it will reassemble into structures that are typical for its composition [22,28]. While in our findings, the cuticle of untreated garlic cloves exhibits a smooth wax film, it is possible that the appearance of wax structures is brought by the combined action of wax oxidation and surface heating during plasma treatment. The increase in the C–O and O–C=O bonds that are seen in high-energy-resolution XPS spectra indicates the appearance of alcohol and ketone functional groups, which both typically imply the presence of platelet structures on epicuticular wax. In fact, the waxes on leaves of onion and leek—both, like garlic, of the genus *Allium*—were shown to form wax platelets, albeit more densely packed, and having serrated edges [22], as opposed to the smooth circular edges that are seen in our case. The size of the structures appears to be roughly comparable. Due to exothermic effects of processes, such as surface neutralization and recombination, the temperature at the sample surface during treatment might rise. For an array of studied plant species, the cuticular wax melting point is typically around 60–80 °C [29]. However, even annealing at temperatures below the wax melting point may be sufficient for wax structure formation, as long as they allow for mobility of the wax molecules [28]. After treatment, the chemically altered waxes may recrystallize in the form of platelets, like the ones seen in our SEM images.

The plasma etching of plant surfaces is possible either by chemical etching through oxidation [23] or by physical sputtering through ion impingement [30]. Let us consider reactive species densities in a low-pressure, inductively coupled, RF oxygen plasma, as used in these experiments. Electron density is typically in the order of 10^16^ m^−3^, while electron temperature is in the order of a few eV. The density of neutral oxygen atoms and ions are in the order of 10^21^ m^−3^ and 10^16^ m^−3^, respectively [31,32]. Etching of the surface would be primarily chemical in nature due to this difference. The key reason why ion density is several orders of magnitude smaller than that of neutral atoms is the difference in the probability for surface loss, which is almost 100% for positively charged ions, while for neutral oxygen atoms on a glass surface, it is below 10^−3^ [33].

As a majority of the plasma treated surface area is only slightly more wrinkled than the untreated one, it appears that only limited etching of the surface occurred during treatment, which is less than what was commonly observed on seeds [34,35,36,37,38], as well as our own observations on unpeeled garlic cloves [24]. The reason lies either in different surface chemistry of the peeled clove when compared to clove peel and seeds, or in the softer treatment conditions due to the lower power density in the present study, as also indicated by the limited oxidation that was recorded by XPS. The distinction between reshaping and etching should also be emphasized, as the first does not necessarily include removal of material form the surface. On the surface of Thuringian mallow seeds, plasma treatment led to a restructuring, or “sharpening”, of the cuticle, which improved the germination parameters. However, longer exposure times also led to surface erosion and cracking, followed by weaker germination improvement [39,40].

Regarding biological responses, the rapid WU during imbibition is the first phase of germination, and it is required for its successful start [41]. For plasma treated seeds, WU typically increases with increased reactive species dose, as exemplified by an increase in the treatment power or time [5,16,42], while other studies found that the increase in seed WU after plasma treatment was comparable, regardless of the treatment power [10,43]. However, in our results, WU increases with increased working pressure, which is associated with a lower plasma density, and thereby a lower reactive species dose (Figure 5). When studying the effect of plasma treatment on surfaces, the effect on both surface chemistry and morphology must be considered. In our experiments, the modest increase in surface oxygen content, which does not significantly differ between treatments, translates to an increase in wettability. In addition, plasma also affects the morphology of the cuticle, which normally acts as a hydrophobic barrier. In seeds, etching of the surface can minimize this water-repellant effect, which increases WU through increased permeability and it is considered to be one of the reasons for germination improvement after plasma treatment [26]. However, with increasing treatment power or time, WU might reach a peak value and decline after further dose increases [3,44]. Further, even when permeability and hydrophilicity are increased with both treatment power and time, WU does not necessarily differ between treatments [45]. Both observations indicate that WU does not solely depend on surface etching, but also on other factors. Despite a more wrinkled clove surface after treatment, we see no etching or loss of structural integrity of the cuticle and, therefore, cannot relate the WU increase to these effects. We further cannot attribute the differences in WU increase after treatment to water loss during treatment. Fresh garlic cloves contain approximately 65% of water [1], and we can expect some loss of water from the clove bulk during plasma treatment, similarly to what was shown for beans [46]. However, the limited degree of etching keeps its protective function against excessive water loss intact [25], and our results confirm that clove mass loss during plasma treatment is minimal and independent of treatment conditions.

The wax structures that arise on the clove surface can also relate to its water affinity. In general, wax crystals on cuticle surfaces are a natural source of hydrophobic properties, and they are often mimicked in artificial material development for this reason [47]. Particularly, structures in the size range of a few hundred nm are typically hydrophobic, imparting water repellent properties on plant surfaces that are covered with such crystals. For example, surface wetting and droplet adhesion of soybean leaves increased after wax platelets were dissolved with surfactants [48]. Further, size and quantity of wax crystals are significantly more important for water affinity than wax chemistry [48], so it is reasonable to assume that the minimal surface oxidation that we observe on cloves is less significant than the extensive crystal formation. As the area that is covered by the wax structures increases with decreasing pressure, the hydrophobic effect the wax structures becomes more pronounced. Thus, while the total WU of the clove increases by oxidation and etching, the effect of the wax platelets rendering the affected areas hydrophobic could explain the inverse relation between WU and reactive plasma species dose.

Several studies on seeds relate shoot and root growth improvement after plasma treatment to increased imbibition due to surface etching or cracking [3,5,44]. However, WU increase does not necessarily translate to germination improvement linearly. The beneficial effects may peak at an optimal dose [10,24,44] for reasons such as damage to the seed material at higher doses. While the effect on WU is thought to be one of the contributing factors to germination improvement after plasma treatment [26], it was shown that plasma affects plant germination and growth, even when eliminating this factor by pre-soaking all seeds [38]. In our results, the increase in root length and number is inversely related to the WU measurements. It is known that a permissive range of water is an essential requirement for the successful germination of plant seeds [41] and, by extension, garlic cloves. In soil, optimal moisture potential exists, below and above which germination is delayed. Water availability and surface-water contact are both important factors, and if water films form on the surface due to hydrophobic properties, such as the nanostructure of wax crystals in our case, germination rate can decrease due to the decreased availability of oxygen to the seed [49]. While plasma treatment of seeds has been previously shown to help seedlings overcome drought stress [11,50,51], it is possible that the higher WU levels that are seen after our treatment are increased above the optimal point, which causes a decrease in the germination rate from the value seen with the 15 Pa treatment.

The effect of oxygen plasma on seed germination and growth is generally described as the action of ROS, which, in addition to seed surface modifications, also activate biochemical pathways within the seeds [52]. However, the exact mechanisms are still largely unknown. Antioxidant enzyme activity increases in roots of plasma treated seeds, which indicates elevated oxidative stress [6], but ROS were also proposed as signaling molecules in plants that may enable successful germination [53]. Exposing seeds to EM field alone can also stimulate germination [54,55]. Further, germination, growth, and yield of onion has previously been improved by exposure to magnetic fields [56]. Our experiments did not find improvement in shoot and root growth after exposure to EM field alone, which indicates that the EM field might be one of several important components in plasma treatment, and the result depends on the characteristics of the used EM field. Exposure to vacuum alone was also found to be insufficient for germination improvement [55].

While ROS are important agents of plasma action on biological materials, it is presently unclear as to whether there is any advantage in treating seeds with pure oxygen plasma as opposed to air or other gas mixtures. Hayashi et al. treated radish sprout seeds with an atmospheric plasma jet, obtaining a more significant increase of total plant length (shoot and root) when using oxygen as the working gas when compared to air [57]. Conversely, Meng et al. observed that wheat shoot and root length increases after atmospheric pressure DBD treatment with air, nitrogen, and argon plasma, relating it to etching and improved WU, but found no enhancement using oxygen [37]. This further illustrates that, in the wide variety of plasma sources and conditions available, it is significant to pick one suitable for treating the specific plant species, or even a specific cultivar.

A garlic clove planted in the field might develop along several paths in response to environmental stimuli [58], and an unchanged or even decreased plant height may, in fact, be desired, as it could indicate that the plant devotes more energy and resources to bulbing than growth of the parts above ground. However, it should be noted that the plant heights were recorded at harvest and, as such, do not give any information regarding early stimulation of growth. However, the variations in dry bulb mass correlate with the improvements in root growth, which were also the highest for the 15 Pa treatment. In plasma treatment of wheat seeds, Roy et al. have similarly correlated plant length increase after medium pressure air/oxygen plasma treatment to a subsequent yield increase [16]. Jiang et al. have also shown that, after low-pressure He plasma treatment, yield increase in the field was preceded by increases in plant height and root length at both the seedling and booting stage [14]. This effect could relate to a general stimulation of plant vigor. As root is the plant organ responsible for nutrient absorption, increased root growth and activity after plasma treatment is linked to increased nutrient uptake [12], which improves plant growth. In particular, when garlic is planted in the spring, it is important that the root system is established quickly and efficiently for the plant to grow properly. By carefully selecting the type of plasma and its parameters, this can be potentially achieved in agricultural practice.

## 5. Conclusions

We have shown that by using inductively coupled, low pressure RF oxygen plasma in a large-scale reactor, we can treat garlic cloves and improve their properties. By using a mild plasma treatment, we have seen slight surface oxidation, but significant morphological changes, including the nanostructuring of epicuticular wax. This is contrary to our previous work on garlic peel surface, where we have observed significant etching and a more pronounced increase in surface oxygen, which is related to the use of a different reactor and plasma parameters. Water uptake into the clove is also modified through the surface changes.

In laboratory germination and field growth, the results of plasma treatment manifest as stimulation of root growth, as well as non-significant increases in shoot length, plant height at harvest, and yield. The observed trends indicate that shoot and root growth can be stimulated by selecting suitable treatment parameters. In particular, the significant acceleration of root growth might hold importance for spring planting of garlic cloves, when efficient root growth is required for successful root system establishment.

The findings warrant further investigation into the effects of oxygen plasma treatment on garlic cloves, particularly on biochemistry and metabolic activity, and how those effects are carried by reactive plasma species and other plasma components from the surface into the bulk of the clove.

## Figures and Tables

**Table 1:** Relative concentration of C 1s sub-peaks [%] after peak fitting.

Treatment	C–C/C–H (~284.8 eV)	C–O (~286.5 eV)	O–C=O (~289.0 eV)
untreated	97.74	1.61	0.66
60 Pa	95.59	2.99	1.43
45 Pa	96.42	1.55	2.04
30 Pa	95.57	1.59	2.84
15 Pa	94.68	3.60	1.72
